# The impact of varying intraoperative oxygen concentrations on lung ultrasound scores and postoperative recovery among elderly patients residing in moderate altitude areas during laparoscopic surgery

**DOI:** 10.3389/fphys.2026.1803883

**Published:** 2026-07-13

**Authors:** Haiyan Yu, Jue Xie, Yeyuan Jin, Xingwen Li, Zhiqin Zhang, Zongzhao He

**Affiliations:** 1 Department of Anesthesiology, Qinghai Provincial People’s Hospital, Qinghai, China; 2 Department of Anesthesiology, surgery and pain management & Key Laboratory of Clinical Science and Research, Zhongda Hospital, Southeast University, Nanjing, Jiangsu, China; 3 Department of Gastrointestinal Surgery, Qinghai Provincial People’s Hospital, Qinghai, China

**Keywords:** elderly patients, fraction of inspired oxygen, laparoscopic, lung ultrasound score, moderate altitude, postoperative pulmonary complications

## Abstract

**Study objective:**

To investigate the impact of different fraction of inspired oxygen (FiO_2_) during laparoscopic surgery on lung ultrasound scores (LUS) and postoperative recovery in elderly patients residing at moderate altitudes, with the objective of determining the optimal intraoperative FiO_2_.

**Methods:**

150 elderly patients residing in areas with an altitude ranging from 2,000 to 3,000 meters for an extended period who underwent laparoscopic surgery were randomly allocated into three groups based on different intraoperative FiO_2_: low-FiO_2_ group (with the FiO_2_ of 30%), medium-FiO_2_ group (with the FiO_2_ of 50%), and high-FiO_2_ group (with the FiO_2_ of 80%). The primary outcome was LUS at 1 hour after tracheal extubation. Secondary outcomes included the incidence of postoperative pulmonary complications (PPCs), the arterial partial pressure of oxygen to fraction of inspired oxygen ratio (PaO_2_/FiO_2_), Serum lactic acid content (LAC), pulmonary dynamic compliance (CDYN) and so on.

**Main results:**

The final intention-to-treat (ITT) analysis included 150 patients, and the per-protocol analysis involved 131. The results indicated that among the three groups, Group M exhibited the best performance in relation to the primary indicator (LUS) and the crucial secondary indicators (PPCs, PaO_2_/FiO_2_, LAC and length of hospital stay). Based on the ITT analysis, for Group L and Group H, certain indicators (CDYN, PaO_2_/FiO_2_ and LAC) presented significant differences during the operation or at 1 hour post - operation. However, the majority of indicators (LUS, PPCs, PaO_2_/FiO_2_, LAC, neutrophils and length of hospital stay) showed no significant differences at 48 hours after the operation.

**Conclusion:**

Our study suggested that for elderly long-term residents at moderate altitudes, a 50% FiO_2_ during laparoscopic surgery yields the most favorable lung ultrasound outcomes and postoperative recovery, compared to 30% and 80%.

**Clinical Trial Registration:**

http://www.chictr.org.cn/, identifier ChiCTR250011206.

## 1 Introduction

Over the past two decades, laparoscopic surgery has emerged as the standard treatment modality for numerous gastrointestinal surgical procedures for it has advantages such as less bleeding and faster recovery (Liu et al., 2022; Yoh et al., 2022; Tian et al., 2024). However, the establishment of pneumoperitoneum during surgical operations and the adoption of specific body positions (e.g., the Trendelenburg position) can result in an elevation of the diaphragm, an augmentation of thoracic pressure, and a decrease in functional residual capacity and as a consequence, this can induce atelectasis both during and after the surgical procedure (Acosta et al., 2018; Brandão et al., 2019; Shono et al., 2020). Impaired gas exchange, hypox-emia, and reduced functional residual capacity (FRC) caused by atelectasis may increase the risk of PPCs, which leads to higher morbidity and mortality, as well as prolonged hospitalization (Mini et al., 2021; Park et al., 2021; Ferrando et al., 2025).

The characteristics of the plateau environment include low atmospheric pressure and low partial pressure of oxygen, which can result in a decrease in arterial partial pressure of oxygen and a reduction in maximal oxygen uptake (Parodi et al., 2023; Wang et al., 2025). Owing to the age-related decline in respiratory system function, such as diminished lung elasticity, weakened respiratory muscle strength, and pulmonary vascular remodeling, elderly patients face a higher risk of PPCs (Miskovic and Lumb, 2017; Castro et al., 2024). In clinical practice, a higher fraction of FiO_2_ is typically employed to prevent intraoperative hypoxemia. Nevertheless, this may expedite the elimination of nitrogen in the alveoli, thereby elevating the risk of atelectasis due to absorption (Zeng et al., 2022). Therefore, determining the ideal FiO_2_ during surgery for elderly patients who have long-term residency on the plateau, which can maintain adequate oxygenation while optimizing the protection of lung function, is one of the pressing issues to be addressed in the perioperative management of plateau patients.

In comparison with chest computed tomography, portable lung ultrasound (LU) can be conducted at the bedside, and it has been extensively demonstrated to be non-invasive, radiation-free, rapid, sensitive, and highly repeatable; LU can sensitively and dynamically evaluate alterations in lung ventilation and exhibits superior diagnostic efficacy for atelectasis compared to chest X-ray (Lichtenstein et al., 2004; Huang et al., 2025). Therefore, LU may provide certain technical support for accurately quantifying the impact of FiO_2_ on the lungs and guiding postoperative rehabilitation.

This study was designed to assess the impacts of different intraoperative FiO_2_ levels (30%, 50%, and 80%) on LUS and postoperative recovery among elderly patients undergoing laparoscopic surgery in moderate-altitude areas ranging from 2000 to 3000 meters (Bärtsch et al., 2008; Chen et al., 2025), with the aim of offering a certain reference foundation for the selection of the optimal intraoperative FiO_2_ for elderly patients in plateau regions.

## 2 Materials and methods

### 2.1 study design

This randomized double-blinded controlled trial was conducted at Qinghai Provincial People’s Hospital. The study protocol was approved by the Ethics Committee of Qinghai Provincial People’s Hospital (No. (2023) -41) and registered in the Chinese Clinical Trial Register (ChiCTR2500112062, http://www.chictr.org.cn/). Written informed consent was obtained from all participants or their legal representatives before recruitment. This study complies with the Declaration of Helsinki and adhered to the 2010 Consolidated Standards of Reporting Trials (CONSORT) (Schulz et al., 2010).

### 2.2 Participants

The investigators screened eligible patients the day before surgery (or on Friday if they underwent surgery the following Monday). Patients who met the following criteria were included: aged 65–80 years, ASA status I-III, scheduled to laparoscopic radical resection of gastric or colon tumor and long-term residents in the moderate altitude areas of Qinghai Province (residing in the moderate-altitude areas of Qinghai Province at an altitude of 2000 to 3000 meters for at least one year up to the day of surgery). Patients who met any of the following criteria were excluded: preoperative coexisting respiratory system diseases (chronic obstructive pulmonary disease, acute exacerbation of asthma, acute stage of pneumonia, upper respiratory tract infection), prior history of pulmonary surgery, having underwent mechanical ventilation within the past two weeks, cognitive impairment or inability to communicate and unwillingness to participate in the research.

According to existing research, the ideal duration of laparoscopic surgery should not exceed 240 minutes (Park et al., 2023). Nevertheless, the medical level in Qinghai Province lags behind the national average, leading to a longer average operation time for surgeons and the pneumoperitoneum duration of most successful laparoscopic gastrointestinal surgeries ranges from 3–4 hours. Taking the actual situation into account and aiming for sample homogeneity as much as possible, we have set the pneumoperitoneum duration at 3–4 hours, and cases that do not meet this criterion will be eliminated. Ultimately, patients who met any of the following criteria were eliminated: the duration of pneumoperitoneum is less than 3 hours or more than 4 hours, massive intraoperative hemorrhage (blood loss exceeding 600 ml) and intraoperative blood oxygen saturation (SpO_2_) is lower than 92%, necessitating adjustment (increasing the FiO_2_) to normal SpO_2_.

### 2.3 Randomization and blinding

This study included 150 patients who underwent laparoscopic radical resection of gastric or colon tumor under general anesthesia. Participants were numbered sequentially based on their enrollment order. A nurse used IBM SPSS Statistics 27 to generate random numbers and randomly allocate participants to one of the three groups in a 1:1:1 ratio. The randomization sequence was generated and placed in sequentially numbered sealed radiopaque envelopes. Once the investigator confirmed eligibility, the envelopes were opened sequentially and participants were assigned to their respective groups by the designated nurse who performed numerical randomization. This study was assessor-blinded. The patients, the physician who conducts the lung ultrasound examination, researchers who performed data collection postoperative, and clinical staff in the postanesthesia care unit (PACU) or ward were blinded to group allocation throughout the study.

### 2.4 Intervention

#### 2.4.1 Preparations subsequent to entering the operating room

Upon entering the operating room, peripheral venous access was established for all patients, and routine monitoring of electrocardiogram, non - invasive blood pressure, and SpO_2_ was conducted. On the premise of conducting Allen’s test to evaluate the blood supply of the radial artery, the left radial artery is preferably selected for puncture and catheterization. Once the puncture is successful, blood gas analysis would be promptly carried out, and invasive arterial blood pressure would be monitored. Finally, the patients were administered nasal cannula oxygen at a flow rate of 3 L/min; simultaneously, a designated physician conducted a preoperative LU examination and documented the LUS.

#### 2.4.2 Intraoperative management

General anesthesia was standardized, and no premedication was administered. Anesthesia was induced intravenously with midazolam (0.03–0.05 mg/kg), propofol (1.5–2.5 mg/kg), sufentanil (0.3–0.5 μg/kg), and rocuronium (0.6–0.9 mg/kg). Anesthesia depth was adjusted by target-controlled infusion of propofol and inhalation of a sevoffurane/oxygen/air mix to maintain a bispectral index value between 40 and 60. The remifentanil infusion rate was adjusted based on the mean arterial pressure and heart rate (within 20% of the baseline values). All patients received treatment with lung-protective ventilation strategies and categorized into distinct groups according to the different FiO_2_: Group L (with the FiO_2_ of 30%), Group M (with the FiO_2_ of 50%), and Group H (with the FiO_2_ of 80%). Arterial blood was drawn from the patients for blood gas analysis at 1 hour and 3 hours after mechanical ventilation, respectively.

#### 2.4.3 Postoperative management

From the moment of extubation in the PACU until two days after the patient’s return to the ward, the patients would continue to receive nasal cannula oxygen at a flow rate of 2 L/min. At 1 hour subsequent to extubation, a LU examination would be conducted by a designated physician and documented the LUS, and then arterial blood would be drawn from the patient for blood gas analysis. At the postoperative hour 48, in addition to conducting a lung ultrasound and collecting arterial blood for blood gas analysis, venous blood was also collected for a complete blood count.

#### 2.4.4 Lung ultrasound examination

LU examinations were conducted by one of the three designated and experienced physicians who were not participating in the study, utilizing a portable ultrasound device (Mindray M10 Exp, Shenzhen, China) and a 6.0 MHz convex array ultrasound probe to evaluate the lung conditions. All LU examinations were conducted by designated physicians who were blinded to the group assignment. The physicians examined six regions in each hemithorax (a total of 12 regions), encompassing the anterior, middle, and posterior regions of the lungs. Each region was inspected, classified, and scored by the physicians (for specific calculation methods, refer to “*2.5 Outcomes*”).

#### 2.4.5 Lung-protective ventilation strategies (LPVS)

LPVS is a respiratory support strategy designed to maintain adequate oxygenation of the body while preventing excessive alveolar expansion and collapse. This approach serves to protect and enhance lung function, as well as reduce the incidence of ventilator-induced lung injury (VILI) and PPCs (Neto et al., 2014). The primary methods encompass small tidal volume, individualized moderate positive end-expiratory pressure (PEEP), and intermittent lung recruitment (Bluth et al., 2019). Recent research has indicated that, when compared to a fixed PEEP of 5 cm H_2_O, individualized PEEP guided by electrical impedance tomography (EIT) does not lead to a reduction in the incidence of PPCs (Wang et al., 2026). In view of the technical challenges associated with providing personalized PEEP during actual operation and the need to ensure the homogeneity of the samples to the greatest extent possible, we have balanced the situation and set the PEEP at 5 cm H_2_O.

All patients were ventilated using the volume-controlled ventilation (VCV) mode. The tidal volume was set at 6–8 ml/kg, the positive end-expiratory pressure (PEEP) was maintained at 5 cm H_2_O, and the respiratory rate was set at 12–16 breaths per minute. Given that carbon dioxide was utilized to establish pneumoperitoneum during the surgical procedure, on the premise of permissive hypercapnia, the end-tidal partial pressure of carbon dioxide (PaCO_2_) is maintained within the range of 35–60 mmHg (Joe et al., 2023). Lung recruitment maneuvers (30–40 cm H_2_O peak inspiratory pressure and 20 cmH_2_O PEEP for 30s (Bluth et al., 2019; Martinsson et al., 2021) were carried out once at the commencement of mechanical ventilation, hourly during mechanical ventilation, and prior to the termination of mechanical ventilation.

### 2.5 Outcomes

The primary outcome was the LUS at 1 hour after tracheal extubation (T_3_). The main secondary outcomes included: the incidence of PPCs; PaO_2_/FiO_2_ and LAC prior to the administration of anesthesia (T_0_), at the 1-hour mark of mechanical ventilation (T_1_), the 3-hour mark of mechanical ventilation (T_2_), T_3_ and the postoperative hour 48(T_4_); LUS at T_0_ and T_4_; CDYN at the 5-minute mark of mechanical ventilation (T’_0_), T_1_ and T_2_. Other pre-specified secondary outcomes included: blood glucose, heart rate, mean arterial pressure and PaCO_2_ at T_0_, T_1_, T_2_, T_3_ and T_4_; duration of mechanical ventilation and pneumoperitoneum; intraoperative fluid infusion volume and blood loss; white blood cell (WBC) and Neutrophil at T_4_ and the postoperative hospital stay. The calculation method of the LUS is as follows: Each hemithorax was examined in six regions (for a total of 12 regions) by the physician, encompassing the anterior, middle, and posterior regions of the lungs. The physician examine each area and categorize it into one of the four ultrasound aeration patterns: Normal aeration (N), the presence of lung sliding and/or lung pulsation, accompanied by A-lines, or fewer than two isolated B-lines per intercostal space; Moderate loss of lung aeration (B1), multiple B-lines distributed at intervals, with more than three B-lines per intercostal space; Severe loss of lung aeration (B2), multiple fused B-lines (with or without subpleural consolidation); Lung aeration (C), the presence of a tissue-like pattern, with or without an air bronchogram. Aggregate the scores obtained from the lung examination of each area, where N = 0, B1 = 2, B2 = 2, C = 3 to derive the final LUS score (ranging from 0 to 36 points). PPCs was defined as the manifestation of the following conditions within 7 days subsequent to surgery: atelectasis detected via computed tomography or chest X-ray examination, pneumonia diagnosed according to the criteria established by the Centers for Disease Control and Prevention of the United States, acute respiratory distress syndrome defined in line with the Berlin Consensus definition and the verify pulmonary aspiration by the clear clinical history or radiological evidence (Abbott et al., 2018).

### 2.6 Sample size calculation

This study adopted a three-group prospective controlled design and the LUS measured at 1 hour after extubation served as the primary efficacy indicator. The overall differences among the three groups are assessed through Kruskal-Wallis H test. Based on pre-trial data, the type I error probability (α) was set at 0.05 (two - sided), the statistical power (1 - β) was set at 90%, and the mean values of each group were 5.6, 4.4, and 6.0 respectively; the standard deviations were estimated to be 1.36, 2.06, and 1.10 respectively. According to the calculation by PASS 15 (NCSS, Kaysville, UT, USA), 40 cases are required for each group, amounting to a total of 120 cases for the three groups. Taking a 20% dropout rate into account, a total of 150 cases are needed for the three groups.

### 2.7 Statistical analysis

Efficacy analyses were based on the intention-to-treat (ITT) population for all endpoints. In addition, sensitivity analyses using the per-protocol population were performed for the LUS and the incidence of PPCs. All data were checked for normal distribution using the Kolmogorov-Smirnov test. Continuous data are presented as mean ± standard deviation (SD), confidence intervals (CIs) for normally distributed variables and medians with CIs for nonnormally distributed data. Categorical variables were summarized using proportions with CIs. Continuous variables were analyzed using analysis of variance or Kruskal-Wallis H test. Categorical variables were compared using the Pear-son’s χ2 test or Fisher’s exact test, as appropriate. In addition, we utilized generalized estimating equations (GEEs) with robust standard error estimates to address the repeated measurements of LUS, PaO_2_/FiO_2_, and LAC, where the group was a fixed effect, and time and the interaction between the group and time were random effects.

Pairwise comparisons between the three groups were considered exploratory analyses. The significance level and confidence interval (CI) employed for inter-group comparisons were set at 0.05 and 95%, respectively. In the event that significant differences were detected among the groups, to control for type I error inflation due to multiple comparisons, a Bonferroni correction was applied. Accordingly, the significance level and CI for each pairwise comparison was adjusted to 0.017 (0.05/3) and 98.3%. Statistical analyses were performed using IBM SPSS version 27 or GraphPad Prism 10.0.

## 3 Results

### 3.1 Study population

**Figure 1:**
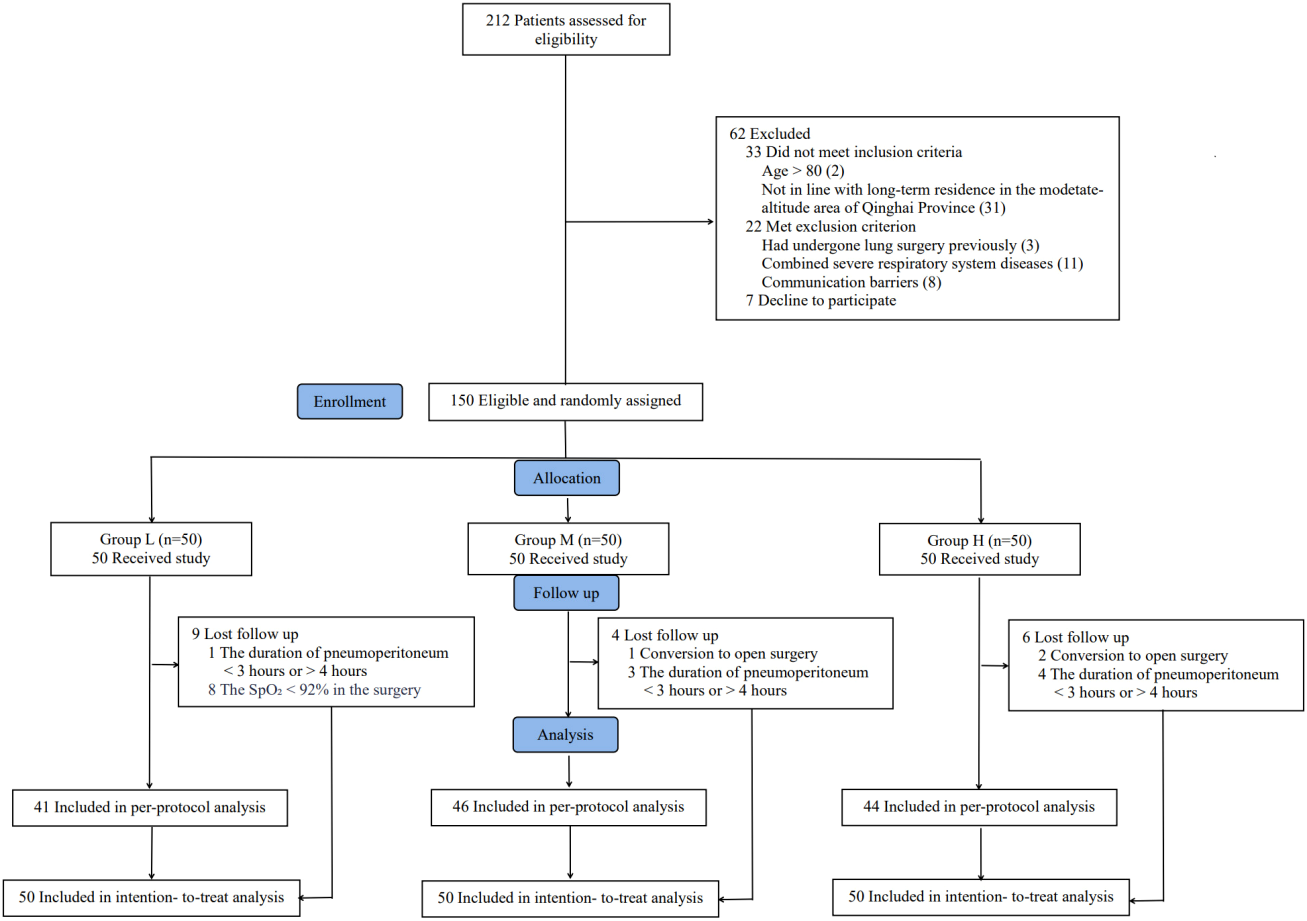
CONSORT diagram for the study. Group L, the group with the intraoperative fraction of inspired oxygen of 30%; Group M, the group with the intraoperative fraction of inspired oxygen of 50%; Group H, the group with the intraoperative fraction of inspired oxygen of 80%.

A total of 212 patients were assessed for eligibility, and 150 patients were randomized. The final ITT analysis incorporated 150 patients, while the per-protocol analysis encompassed 131 patients (**Figure 1**). Overall, the patient demographics and surgical and anesthetic characteristics were balanced among the three groups (**Table 1**).

**Table 1:** Demographic and clinical characteristics at baseline.

Characteristic	Group L (n=50)	Group M (n=50)	Group H (n=50)
Age, mean (SD), y	69.0 (5.4)	67.2 (4.1)	67.5 (5.6)
Gender			
Men, n (%)	26 (52.0)	28 (56.0)	32 (64.0)
Women, n (%)	24 (48.0)	22 (44.0)	18 (36.0)
BMI, mean (SD), kg/m^2^	24.6 (3.5)	23.8 (3.0)	24.1 (3.2)
ASA status, n (%)			
II	44 (88.0)	40 (80.0)	41 (82.0)
III	6 (12.0)	10 (20.0)	9 (18.0)
Comorbidity, n (%)			
Hypertension	16 (32.0)	21 (42.0)	24 (48.0)
CAD	10 (20.0)	13 (26.0)	11 (22.0)
DM	6 (12.0)	10 (20.0)	9 (18.0)
Types of surgical procedures, n(%)			
Radical gastrectomy	22 (44.0)	18 (36.0)	24 (48.0)
Radical resection of colon tumors	28 (56.0)	32 (64.0)	26 (52.0)

Data are presented as mean (standard deviation) or number (percentage).

Group L, the group with the intraoperative fraction of inspired oxygen of 30%; Group M, the group with the intraoperative fraction of inspired oxygen of 50%; Group H, the group with the intraoperative fraction of inspired oxygen of 80%; BMI, body mass index; ASA, American Society of Anesthesiologists; CAD, coronary artery disease; DM, diabetes mellitus; SD, standard deviation.

### 3.2 The lung ultrasound score (LUS)

The baseline was balanced at T_0_ among the three groups (**Table 2**).

**Table 2:** Perioperative characteristics and some indicators among the three groups.

Parameter	Group L (n=50)	Group M (n=50)	Group H (n=50)
BG, median (IQR), mmol/L			
T_0_	6.1 (5.0, 7.2)	5.7 (5.1, 7.2)	6.1 (5.3, 7.1)
T_1_	6.6 (5.2, 7.4)	6.3 (5.6, 7.1)	6.7 (5.8, 7.4)
T_2_	6.7 (5.6, 7.9)	6.7 (5.9, 7.8)	6.9 (6.1, 7.9)
T_3_	7.1 (6.1, 7.8)	6.8 (6.1, 8.3)	7.2 (6.2, 8.2)
T_4_	7.0 (5.7, 7.9)	6.9 (6.0, 8.1)	7.3 (6.3, 8.0)
HR, median (IQR), bpm			
T_0_	61.0 (57.5, 72.0)	66.0 (57.0, 73.0)	62.5 (56.5, 72.0)
T_1_	72.0 (62.0, 84.0)	67.0 (62.0, 73.0)	68.0 (64.0, 76.0)
T_2_	72.0 (67.0, 86.5)	71.0 (65.0, 82.0)	74.0 (67.0, 80.0)
T_3_	72.0 (65.0, 80.0)	69.5 (63.0, 75.0)	76.0 (69.0, 80.0)
T_4_	80.5 (68.0, 87.0)	76.5 (69.5, 83.5)	79.0 (66.5, 85.0)
MAP, median (IQR), mmHg			
T_0_	88.0 (78.5, 100.0)	89.0 (81.0, 99.0)	95.0 (80.5, 100.0)
T_1_	78.0 (72.5, 84.0)	78.0 (72.0, 89.0)	77.0 (71.5, 82.5)
T_2_	88.0 (80.5, 98.5)	87.0 (81.0, 94.0)	88.5 (80.0, 95.5)
T_3_	90.0 (80.0, 97.0)	85.8 (79.0, 96.0)	92.0 (82.5, 97.0)
T_4_	97.0 (83.0, 107.0)	91.5 (79.0, 98.0)	95.0 (81.5, 105.0)
PaCO_2_, median (IQR), mmHg			
T_0_	35.5 (32.7, 38.0)	35.2 (33.4, 37.0)	37.1 (34.1, 39.0)
T_1_	47.0 (42.5, 50.4)	46.0 (41.1, 49.1)	48.0 (45.8, 49.2)
T_2_	52.8 (48.6, 56.1)	52.8 (48.0, 56.1)	53.5 (51.0, 55.2)
T_3_	41.1 (39.1, 44.8)	39.7 (36.7, 44.5)	43.0 (39.5, 45.5)
T_4_	42.5 (37.6, 46.7)	37.5 (35.7, 42.5)	43.2 (38.5, 46.5)
Duration of mechanical ventilation, median (IQR), h	5.2 (4.5, 6.5)	5.2 (4.5, 6.0)	5.8 (5.0, 6.0)
Duration of pneumoperitoneum, median (IQR), h	3.5 (3.1, 4.0)	3.4 (3.0, 3.7)	3.5 (3.1, 3.8)
Intraoperative fluid infusion volume, mean (SD), L	2.0 (1.3, 2.7)	2.2 (1.3, 2.8)	2.2 (1.4, 2.9)
Intraoperative blood loss, median (IQR), ml	100.0 (50.0, 200.0)	100.0 (50.0, 200.0)	100.0 (50.0, 200.0)
Baseline Indicators			
LUS at T_0_, median (IQR)	1.0 (0.0, 2.0)	1.0 (0.0, 3.0)	1.0 (0.0, 2.0)
PaO2 /FiO2 at T_0_, mean (SD), mmHg	283.5 (17.1)	286.3 (20.6)	279.8 (15.7)
CDYN at T’_0_, mean (SD), ml/cmH2O	40.2 (5.4)	39.6 (7.7)	38.5 (6.7)
LAC at T_0_, median (IQR), mmol/L	1.3 (1.0, 1.6)	1.0 (0.9, 1.5)	1.1 (0.9, 1.5)

Data are presented as median (interquartile range) or mean (standard deviation).

Group L, the group with the intraoperative fraction of inspired oxygen of 30%; Group M, the group with the intraoperative fraction of inspired oxygen of 50%; Group H, the group with the intraoperative fraction of inspired oxygen of 80%; BG, blood glucose; HR, heart rate; MAP, mean arterial pressure; PaCO_2_, carbon dioxide partial pressure; LUS, lung ultrasound score; PaO_2_ /FiO_2_, the arterial partial pressure of oxygen to fraction of inspired oxygen ratio; CDYN, pulnonary dynamic compliance; LAC, serum lactic acid content; T_0_, prior to the administration of anesthesia; T’_0_, the 5-minute mark of mechanical ventilation; T_1_, the 1-hour mark of mechanical ventilation; T_2_, the 3-hour mark of mechanical ventilation; T_3_, 1 hour after tracheal extubation; T_4_, the postoperative hour 48; IQR, interquartile range;SD, standard deviation.

**Figure 2:**
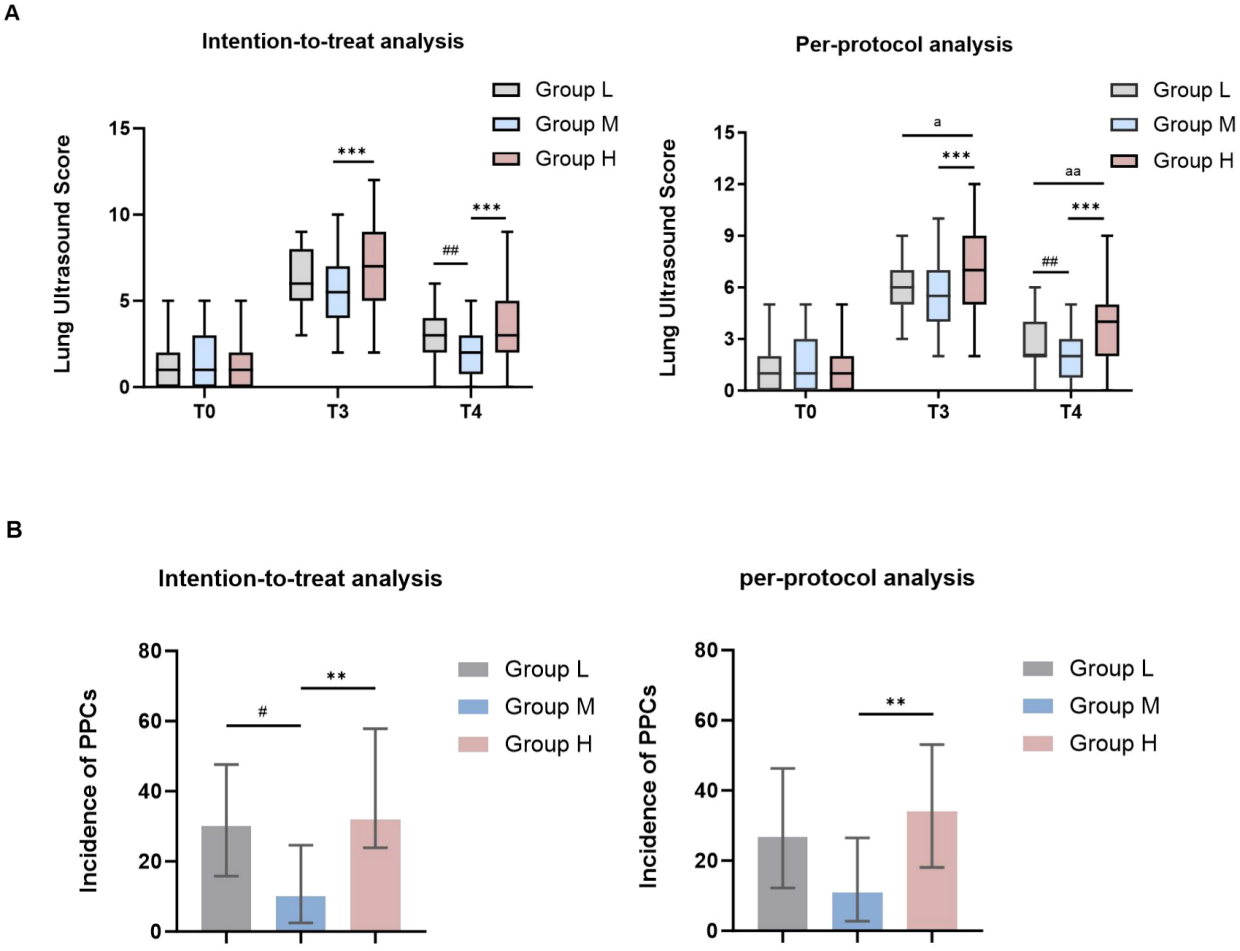
Comparison of some indicators among the three groups. **(A)** Lung Ultrasound Score; **(B)** Incidence of PPCs. Error bars represent the range from the maximum to the minimum value **(A)** and the 98.3% confidence interval **(B)**; The symbol “#” indicates a significant difference between Group L and Group M; The asterisk (*) indicates a significant difference between Group M and Group (H) The letter “a” indicates a significant difference between Group L and Group (H) PPCs, postoperative pulmonary complications within 48 hours after surgery; Group L, the group with the intraoperative fraction of inspired oxygen of 30%; Group M, the group with the intraoperative fraction of inspired oxygen of 50%; Group H, the group with the intraoperative fraction of inspired oxygen of 80%; T0, prior to the administration of anesthesia; T3, 1 hour after tracheal extubation; T4, the postoperative hour 48.

At T_3_, while the ITT analysis of the LUS indicated a statistically significant difference solely for Group M over Group H (median difference, -2.0; 98.3% CI, -3.0 to -1.0; *P* < 0.001), the numerical comparison indicated that Group M performed the best. The per-protocol analysis yielded a different result for the comparison between Group L and Group H, showing Group L to have better performance (median difference, -1.0; 98.3% CI, -2.0 to 0.0; *P* = 0.012) (**Table 3**, **Figure 2**).

**Table 3:** Comparison of the lung ultrasound score and incidence of PPCs.

Key Parameter	Group L	Group M	Group H
Intention-to-treat	n=50	n=50	n=50
LUS at T_3_, median (98.3% CI)	6.0 (6.0 to 7.0)	5.5 (5.0 to 6.0)	7.0 (7.0 to 8.0)
Absolute differences, median (98.3% CI)^a^			
Compared with Group L *P* value	NA	1.0 (0.0 to 2.0) *P =* 0.019^c^	-1.0 (-2.0 to 0.0) *P =* 0.022^c^
Compared with Group M *P* value	NA	NA	-2.0 (-3.0 to -1.0) *P* < 0.001^b^
LUS at T_4_, median (98.3% CI)	3.0 (2.0 to 3.0)	2.0 (1.0 to 2.0)	3.0 (2.0 to 5.0)
Absolute differences, median (98.3% CI)^a^			
Compared with Group L *P* value	NA	1.0 (0.0 to 2.0) *P =* 0.002^b^	-1.0 (-2.0 to 0.0) *P =* 0.061
Compared with Group M *P* value	NA	NA	-2.0 (-3.0 to -1.0) *P* < 0.001^b^
Incidence of PPCs, IR (98.3% CI), %d	30.0 (15.8 to 47.6)	10.0 (2.5 to 24.6)	32.0 (23.9 to 57.8)
Absolute differences, IR (98.3% CI), %^e^			
Compared with Group L *P* value	NA	20.0 (1.5 to 38.5) *P =* 0.012^b^	-2.0 (-24.1 to 20.1) *P =* 0.829
Compared with Group M *P* value	NA	NA	-22.0 (-40.7 to -3.3) *P =* 0.007^b^
*Per-protocol*	n=41	n=46	n=44
LUS at T_3_, median (98.3% CI)	6.0 (6.0 to 7.0)	5.5 (4.5 to 6.0)	7.0 (6.0 to 9.0)
Absolute differences, median (98.3% CI)^a^			
Compared with Group L *P* value	NA	1.0 (0.0 to 2.0) *P =* 0.049^c^	-1.0 (-2.0 to 0.0) *P =* 0.012^b^
Compared with Group M *P* value	NA	NA	-2.0 (-3.0 to -1.0) *P* < 0.001^b^
LUS at T_4_, median (98.3% CI)	2.0 (2.0 to 3.0)	2.0 (1.0 to 2.0)	4.0 (3.0 to 5.0)
Absolute differences, median (98.3% CI)^a^			
Compared with Group L *P* value	NA	1.0 (0.0 to 2.0) *P =* 0.007^b^	-1.0 (-2.0 to 0.0) *P =* 0.008^b^
Compared with Group M *P* value	NA	NA	-2.0 (-3.0 to -1.0) *P* < 0.001^b^
Incidence of PPCs, IR (98.3% CI), %d	26.8 (12.2 to 46.3)	10.9 (2.8 to 26.5)	34.1 (18.1 to 53.1)
Absolute differences, IR (98.3% CI), %^e^			
Compared with Group L *P* value	NA	16.0 (-3.9 to 35.8) *P* = 0.055	-7.3 (-31.0 to 16.5) *P =* 0.468
Compared with Group M *P* value	NA	NA	-23.2 (-43.5 to -2.9) *P =* 0.008^b^

Data are presented as median (98.3% confidence interval) or incidence rate (98.3% confidence interval).

Group L, the group with the intraoperative fraction of inspired oxygen of 30%; Group M, the group with the intraoperative fraction of inspired oxygen of 50%; Group H, the group with the intraoperative fraction of inspired oxygen of 80%; LUS, lung ultrasound score; T_3_, 1 hour after tracheal extubation; T_4_, the postoperative hour 48; PPCs, postoperative pulmonary complications within 48 hours after surgery; IR,incidence rate; CI, confidence interval.

^a^ The median difference of the confidence interval was calculated by the Hodges-Lehmann estimation method. Median differences are ‘left column compared with (minus) top row’.

^b^ This P value ≤ 0.017 (level of significance).

^c^ This P value is above the prespecified threshold (level of significance) of P = 0.017.

^d^ The Bonferroni adjusted 98.3% confidence interval for proportions were calculated using Clopper-Pearson method with binom test.

^e^ The 98.3% confidence interval of absolute difference was calculated using the Newcombe-Wilson method; Absolute differences are ‘left column compared with (minus) top row’.

At T_4_, the ITT analysis of the LUS revealed significant differences between Group L and Group M and between Group M and Group H, with Group M performing better in both comparisons (median difference, 1.0; 98.3% CI of median, 0.0 to 2.0; *P* = 0.002 and median difference, -2.0; 98.3% CI, -3.0 to -1.0; *P* < 0.001). However, it showed no significant difference between Group L and Group H. In contrast, the per-protocol analysis showed a statistically significant difference between Group L and Group H, with Group L demonstrating better performance (median difference, -1.0; 98.3% CI of median, -2.0 to 0.0; *P* = 0.008) (**Table 3**, **Figure 2**).

### 3.3 Secondary outcomes analyses

#### 3.3.1 The incidence of postoperative pulmonary complications (PPCs)

While the ITT analysis showed a lower incidence of PPCs (%) in Group M compared to both Groups L (absolute difference, -20.0; 98.3% CI, -38.5 to -1.5; *P* = 0.012) and Group H (absolute difference, -22.0; 98.3% CI, -40.7 to -3.3; *P* = 0.007), the per-protocol analysis revealed no significant difference in PPCs incidence between Group M and Group L (**Table 3**, **Figure 2**).

#### 3.3.2 The arterial partial pressure of oxygen to fraction of inspired oxygen ratio (PaO_2_/FiO_2_)

The baseline was balanced at T_0_ among the three groups (**Table 2**).

When Group L is compared with Group M, there is only a statistically significant difference in PaO_2_/FiO_2_ (mmHg) at T_2_, and Group M performing better (mean difference, -11.3; 98.3% CI, -21.7 to -0.8; *P* = 0.010) (**Table 4**).

**Table 4:** Comparison of the other indicators among the three groups.

Parameter	Group L (n=50)	Group M (n=50)	Group H (n=50)
PaO2 /FiO2 at T_1_, mean (98.3% CI), mmHg	243.6 (235.0 to 252.2)	238.6 (232.5 to 244.6)	232.7 (226.3 to 239.1)
Absolute differences, mean (98.3% CI), mmHg^a^			
Compared with Group L *P* value	NA	3.4 (-7.1 to 13.8) *P =* 0.433	10.9 (0.4 to 21.4) *P =* 0.014^b^
Compared with Group M *P* value	NA	NA	7.5 (-1.3 to 16.3) *P =* 0.040^c^
PaO2 /FiO2 at T_2_, mean (98.3% CI), mmHg	222.4 (215.8 to 229.0)	234.4 (225.4 to 243.5)	205.2 (198.1 to 239.1)
Absolute differences, mean (98.3% CI), mmHg^a^			
Compared with Group L *P* value	NA	-11.3 (-21.7 to -0.8) *P =* 0.010^b^	17.2 (7.7 to 26.7) *P* < 0.001^b^
Compared with Group M *P* value	NA	NA	28.4 (17.7 to 39.2) *P* < 0.001^b^
PaO2 /FiO2 at T_3_, mean (98.3% CI), mmHg	240.4 (230.6 to 250.3)	252.4 (243.2 to 261.5)	231.4 (222.6 to 240.1)
Absolute differences, mean (98.3% CI), mmHg^a^			
Compared with Group L *P* value	NA	-10.7 (-23.2 to 2.1) *P =* 0.045^c^	9.1 (-3.9 to 22.0) *P =* 0.091
Compared with Group M *P* value	NA	NA	19.8 (7.8 to 31.8) *P* < 0.001^b^
PaO2 /FiO2 at T_4_, mean (98.3% CI), mmHg	248.3 (238.2 to 258.4)	261.0 (251.1 to 270.9)	240.2 (230.2 to 250.2)
Absolute differences, mean (98.3% CI), mmHg^a^			
Compared with Group L *P* value	NA	-12.9 (-26.4 to 0.5) *P =* 0.022^c^	8.1 (-5.9 to 22.1) *P =* 0.162
Compared with Group M *P* value	NA	NA	21.0 (7.7 to 34.0) *P* < 0.001^b^
LAC at T_1_, median (98.3% CI), mmol/L	1.2 (1.0 to 1.5)	1.0 (0.8 to 1.1)	0.8 (0.7 to 0.9)
Absolute differences, median (98.3% CI), mmol/L^d^			
Compared with Group L *P* value	NA	0.2 (-0.0 to 0.4) *P =* 0.030^c^	0.3 (0.1 to 0.5) *P* < 0.001^b^
Compared with Group M *P* value	NA	NA	0.1 (0.0 to 0.3) *P =* 0.029^c^
LAC at T_2_, median (98.3% CI), mmol/L	1.8 (1.5 to 2.0)	1.0 (0.8 to 1.6)	0.8 (0.7 to 0.9)
Absolute differences, median (98.3% CI), mmol/L^d^			
Compared with Group L *P* value	NA	0.5 (0.1 to 0.9) *P =* 0.002^b^	0.9 (0.5 to 1.2) *P* < 0.001^b^
Compared with Group M *P* value	NA	NA	0.2 (0.0 to 0.5) *P =* 0.007^b^
LAC at T_3_, median (98.3% CI), mmol/L	1.5 (1.2 to 1.8)	1.0 (0.7 to 1.1)	1.0 (0.9 to 1.1)
Absolute differences, median (98.3% CI), mmol/L^d^			
Compared with Group L *P* value	NA	0.4 (0.1 to 0.7) *P =* 0.002^b^	0.5 (0.3 to 0.7) *P* < 0.001^b^
Compared with Group M *P* value	NA	NA	0.0 (-0.2 to 0.3) *P =* 0.645
LAC at T_4_, median (98.3% CI), mmol/L	1.5 (1.3 to 1.5)	1.0 (0.9 to 1.1)	1.3 (1.1 to 1.5)
Absolute differences, median (98.3% CI), mmol/L^d^			
Compared with Group L *P* value	NA	0.4 (0.2 to 0.7) *P* < 0.001^b^	0.1 (-0.2 to 0.4) *P* = 0.347
Compared with Group M *P* value	NA	NA	-0.3 (-0.5 to -0.1) *P =* 0.001^b^
CDYN at T_1_, mean (98.3% CI), ml/cmH_2_O	28.0 (26.3 to 29.7)	30.5 (28.5 to 32.4)	25.6 (24.1 to 27.1)
Absolute differences, mean (98.3% CI), ml/cmH_2_O^a^			
Compared with Group L *P* value	NA	-2.5 (-5.1 to 0.1) *P =* 0.021^c^	2.4 (0.2 to 4.7) *P =* 0.010^b^
Compared with Group M *P* value	NA	NA	4.9 (2.5 to 7.4) *P* < 0.001^b^
CDYN at T_2_, mean (98.3% CI), ml/cmH_2_O	23.1 (22.0 to 24.2)	24.7 (23.3 to 26.2)	21.5 (20.2 to 22.7)
Absolute differences, mean (98.3% CI), ml/cmH_2_O^a^			
Compared with Group L *P* value	NA	-1.6 (-3.5 to 0.3) *P =* 0.029^c^	1.6 (0.0 to 3.3) *P =* 0.019^b^
Compared with Group M *P* value	NA	NA	3.3 (1.4 to 5.2) *P* < 0.001^b^
WBC at T4, median (98.3% CI), ×10^9^/L	7.4 (6.8 to 7.8)	6.6 (5.5 to 7.3)	8.4 (7.8 to 9.3)
Absolute differences, median (98.3% CI), ×10^9^/L^d^			
Compared with Group L *P* value	NA	1.2 (0.2 to 2.3) *P =* 0.003^b^	-1.2 (-2.1 to -0.2) *P =* 0.004^b^
Compared with Group M *P* value	NA	NA	-2.4 (-3.5 to -1.3) *P* < 0.001^b^
Neutrophil at T_4_, median (98.3% CI), ×10^9^/L	6.1 (4.8 to 7.7)	4.9 (3.5 to 6.2)	6.1 (5.5 to 6.9)
Absolute differences, median (98.3% CI), ×10^9^/L^d^			
Compared with Group L *P* value	NA	1.4 (0.1 to 3.0) *P =* 0.021^c^	-0.1 (-1.6 to 1.3) *P =* 0.780
Compared with Group M *P* value	NA	NA	-1.5 (-2.8 to -0.3) *P =* 0.006^b^
Postoperative hospital stay, median (98.3% CI), day	8.0 (7.0 to 9.0)	6.5 (6.0 to 7.0)	9.0 (8.0 to 9.0)
Absolute differences, median (98.3% CI), day^d^			
Compared with Group L *P* value	NA	1.0 (0.0 to 2.0) *P =* 0.001^b^	-1.0 (-2.0 to 0.0) *P =* 0.089
Compared with Group M *P* value	NA	NA	-2.0 (-3.0 to -1.0) *P <* 0.001^b^

Data are presented as mean (98.3% confidence interval), or median (98.3% confidence interval).

Group L, the group with the intraoperative fraction of inspired oxygen of 30%; Group M, the group with the intraoperative fraction of inspired oxygen of 50%; Group H, the group with the intraoperative fraction of inspired oxygen of 80%; PaO_2_ /FiO_2_, the arterial partial pressure of oxygen to fraction of inspired oxygen ratio; CDYN, pulnonary dynamic compliance; LAC, serum lactic acid content; WBC, white blood cell T_1_, the 1-hour mark of mechanical ventilation; T_2_, the 3-hour mark of mechanical ventilation; T_3_, 1 hour after tracheal extubation; T_4_, the postoperative hour 48; CI, confidence interval.

^a^ Mean differences are ‘left column compared with (minus) top row’.

^b^ This P value ≤ 0.017 (level of significance).

^c^ This P value is above the prespecified threshold (level of significance) of P = 0.017.

^d^ The median difference of the confidence interval was calculated by the Hodges-Lehmann estimation method. Median differences are ‘left column compared with (minus) top row’.

Group L demonstrated a significantly higher PaO_2_/FiO_2_ (mmHg) than Group H during the operation: T_1_ (mean difference, 10.9; 98.3% CI, 0.4 to 21.4; *P* = 0.014) and T_2_ (mean difference, 17.2; 98.3% CI, 7.7 to 26.7; *P* < 0.001), except for T_3_ and T_4_ after the operation, when no significant difference was observed (**Table 4**).

Group M demonstrated a significantly higher PaO_2_/FiO_2_ (mmHg) than Group H at T_2_ (mean difference, 28.4; 98.3% CI, 17.7 to 39.2; *P* < 0.001), T_3_ (mean difference, 19.8; 98.3% CI, 7.8 to 31.8; *P* < 0.001) and T_4_ (mean difference, 21.0; 98.3% CI, 7.7 to 34.0; *P* < 0.001), except for early-stage T_1_ during surgery, when no significant difference was observed (**Table 4**).

#### 3.3.3 Serum lactic acid content (LAC)

The baseline was balanced at T_0_ among the three groups (**Table 2**).

Group L demonstrated a significantly higher LAC (mmol/L) than Group M at T_2_ (median difference, 0.5; 98.3% CI, 0.1 to 0.9; *P* = 0.002), T_3_ (median difference, 0.4; 98.3% CI, 0.1 to 0.7; *P* = 0.002), and T_4_ (median difference, 0.5; 98.3% CI, 0.1 to 0.9; *P* < 0.001), except for early-stage T_1_ during surgery, when no significant difference was observed (**Table 4**).

The LAC (mmol/L) in Group L was higher than that in Group M at T_2_ (median difference, 0.9; 98.3% CI, 0.5 to 1.2; *P* < 0.001) and T_3_ (median difference, 0.5; 98.3% CI, 0.3 to 0.7; *P* < 0.001). However, no statistically significant difference was found between the two groups at the early surgical phase (T_1_) or 48 hours post-surgery (T_4_) (**Table 4**).

While no difference existed at the early surgical phase (T_1_), the LAC (mmol/L) of Group M became significantly higher than those of Group H at T_2_ (median difference, 0.2; 98.3% CI, 0.0 to 0.5; *P* = 0.007). This difference disappeared by one hour after extubation (T3). Subsequently, 48 hours post-surgery (T4), Group M exhibited lower LAC than Group H (median difference, -0.5; 98.3% CI, -0.5 to -0.1; *P* = 0.001) (**Table 4**).

#### 3.3.4 Pulmonary dynamic compliance (CDYN)

The baseline was balanced at T’_0_ among the three groups (**Table 2**). There was no statistically significant difference in CDYN (ml/cmH_2_O) between Group L and Group M (**Table 4**). There was a statistically significant difference in CDYN (ml/cmH_2_O) between group L and group M at the early-stage T_1_ during surgery (mean difference, 2.4; 98.3% CI, 0.2 to 4.7; *P* = 0.010) (**Table 4**). The CDYN (ml/cmH_2_O) in group M was superior to that in group H at both T_1_ (mean difference, 4.9; 98.3% CI, 2.5 to 7.4; *P* < 0.001) and T_2_ (mean difference, 3.3; 98.3% CI, 1.4 to 5.2; *P* < 0.001) (**Table 4**).

### 3.4 Other pre-specified secondary outcomes

There was no statistically significant difference among the three groups in blood glucose, heart rate, mean arterial pressure and PaCO_2_ at T_0_, T_1_, T_2_, T_3_ and T_4_, as well as the duration of mechanical ventilation and pneumoperitoneum, and intraoperative fluid infusion volume and blood loss (**Table 2**).

The pairwise comparisons of WBC in T4 all indicated statistically significant differences, with Group M exhibiting the optimal performance and Group L ranking second (**Table 4**). The pairwise comparison of neutrophil in T4 only revealed a statistically significant difference between Group M and Group H, with Group M being superior (**Table 4**). The pairwise comparisons of the postoperative hospital stay demonstrated no statistically significant difference between Group L and Group H, whereas Group M outperformed in all other comparisons (**Table 4**).

## 4 Discussion

### 4.1 Selection of the study population

Currently, approximately 5 million individuals reside in plateau regions globally, constituting a substantial group worthy of our attention (Tremblay and Ainslie, 2021). The majority of areas in Qinghai Province of China are at medium altitude, and the Qinghai Provincial People’s Hospital is the premier hospital in the province. Consequently, numerous residents in the province opt for this hospital for medical treatment. Additionally, regardless of disparities in gender, region, and the level of the human development index, both cancer and aging are emerging as an increasingly significant global burden (Wang et al., 2025). Therefore, in accordance with local circumstances, we selected elderly patients who have long-term residence at moderate altitude and have undergone laparoscopic surgery for this study, with the aim of providing some evidence for future clinical decision-making. It may also offer a direction and basis for this population to receive more attention and research in the future.

### 4.2 Selection and bases of three different inspired oxygen concentrations as variables

Currently, the majority of studies on the perioperative FiO_2_ have concentrated on populations in non-high-altitude areas and the comparison between low FiO_2_ (approximately 30%) and high FiO_2_ (approximately 80%) (Jiang et al., 2021; Markwei et al., 2023; Nam et al., 2023). Most current studies do not advocate the routine use of high FiO_2_ (80%) during the perioperative period as the preferred strategy; Instead, they believe that low FiO_2_ (30%) may offer more advantages in terms of safety and the potential benefits of hyperoxia (such as a slight reduction in surgical-site infections) should be balanced against the risks (such as PPCs) (Jiang et al., 2021; Douville et al., 2024; Elfeky et al., 2025). The characteristics of the plateau environment, including low atmospheric pressure and low oxygen partial pressure, can result in a decrease in arterial oxygen partial pressure and maximal oxygen uptake (Parodi et al., 2023; Wang et al., 2025). Acute high-altitude exposure can induce hypoxia-related diseases, such as high-altitude pulmonary edema (HAPE) and high-altitude pulmonary hypertension (HAPH) and chronic exposure may also result in pulmonary arterial hypertension and an increased load on the right heart (Pena et al., 2022; Gatterer et al., 2024; Cai et al., 2026). From a pathophysiological perspective, high-altitude hypoxia can disrupt lung lipid metabolism and impair the mitochondrial function of pulmonary microvascular endothelial cells (Hossain and Eckmann, 2023; Xu et al., 2026). Although the physiological structure and genes of residents in the plateau region have adapted to the hypoxic environment (Storz, 2021; Ferraretti et al., 2024), their basic arterial blood oxygen partial pressure and maximal oxygen uptake remain lower than those of individuals residing in plain areas (Richalet et al., 2024). In our clinical practice, it has been found that an intraoperative FiO_2_ of 30% appears to be associated with perioperative hypoxemia (defined by SpO_2_ < 92% during the operation) (Mazzeffi et al., 2021). We made an attempt to elevate the intraoperative FiO_2_ and noted that when it was increased to approximately 50%, the incidence of intraoperative SpO_2_ < 92% seemed to be significantly decreased. Given the particularities of the high-altitude environment, it may not be appropriate to mechanically apply the conclusion—derived from studies in plain areas—that “30% FiO_2_ is superior to 80% FiO_2_.” Furthermore, clinical practice has suggested potential benefits of 50% FiO_2_. Therefore, to obtain more reliable clinical evidence, three levels of FiO_2_ (30%, 50%, and 80%) were ultimately selected as the independent variable.

### 4.3 The decision to utilize LUS as the primary indicator

LU is the technique that has been employed in clinical practice in recent years, primarily utilized in the assessment of critical illness (Mongodi et al., 2021; Yuriditsky et al., 2021). There is a paucity of studies on LUS in the perioperative period, and the studies were mainly concentrated in specific scenarios, such as extracorporeal membrane oxygenation (ECMO) support during lung transplantation or monitoring of postoperative pulmonary complications (Haskins et al., 2021; Reed et al., 2026). Considering that blood gas analysis has been in clinical use since the 1960s (Saidman, 2022) and arterial blood gas analysis serves as an important reference standard for the diagnosis of respiratory failure and metabolic disorders (Byrne et al., 2025), this method has been applied proficiently. LU possesses the advantages of portability, non-invasiveness, absence of radiation, rapid operation, high sensitivity, and strong repeatability, and moreover, its efficiency in the diagnosis of atelectasis is superior to that of chest X-ray (Lichtenstein et al., 2004; Huang et al., 2025). As a beneficial complement to blood gas analysis, LU has been gradually employed in perioperative evaluation (Loi et al., 2024). Therefore, this study ultimately determined to adopt LUS as the primary indicator and blood gas analysis and other indicators as secondary indicators. The objective is to assess the application value of LUS in the perioperative period of elderly residents undergoing surgery in plateau areas, offer some basis for the formulation of clinical strategies, and anticipate further expansion and research in more fields and among more populations in the future.

### 4.4 Exploratory outcomes analyses

#### 4.4.1 The primary and the main secondary outcomes

Both the ITT and per-protocol sensitivity analyses of LUS demonstrated consistent results: Group M was superior to both Group L and Group H. The incidence of PPCs served as an important indicator of postoperative recovery. We also carried out an ITT analysis and per-protocol sensitivity analysis comparison for it, and the results were consistent: in pairwise comparisons, according to the ITT analysis, Group M outperforms Group L and Group H, demonstrating the best performance; according to the PP analysis, although there was no statistical difference between Group L and Group M, Group M showed better numerical values. The PaO_2_/FiO_2_ also exhibit a similar trend. At the early stage of the surgery (T_1_), Group L was superior to Group H, and there was no statistical difference compared to Group M; however, as time elapses, at T_3_ and T_4_, there was no longer a statistically significant difference between Group L and Group H, while Group M was significantly better than Group H. According to the statistical data of LAC, Group H performs optimally during the surgery (T_1_, T_2_). However, at 1 hour after extubation (T_3_), there was no statistical difference between Group M and Group H. By 48 hours after the surgery (T_4_), Group M once again becomes the group with the best performance. In conclusion, among the three groups, Group M exhibits the best performance.

When comparing Group L with Group H, no statistically significant difference in LUS was observed at either T_3_ or T_4_ in the ITT analysis. In contrast, the per-protocol sensitivity analysis suggested better performance in Group L. However, 8 patients eliminated whose intraoperative SpO_2_ could not be maintained above 92% were all in Group L (**Figure 1**). Although we excluded patients with preoperative respiratory system diseases (chronic obstructive pulmonary disease, acute asthma attack, acute pneumonia, upper respiratory tract infection) during the screening process and individuals residing in plateau regions possess a certain degree of tolerance to low partial pressure of oxygen (Storz, 2021; Ferraretti et al., 2024), to guarantee patient safety, for patients with SpO_2_ lower than 92%, we had to increase the inspired oxygen concentration to continue the operation and eliminated them from the group. In fact, an SpO_2_ level below 92% (particularly during anesthesia) may suggest the presence of hypoxemia in the patient, which could be associated with inadequate ventilation, an elevated intrapulmonary shunt, or compromised oxygenation function and simultaneously, intraoperative hypoxemia may elevate the incidence of postoperative complications (Disma et al., 2021; Burnett et al., 2022; Liang et al., 2025). In fact, this process may filter out patients with deviations in lung function within group L and it could potentially be associated with the better performance of Group L in the PP analysis. Furthermore, both ITT and PP analyses of PPCs showed no statistically significant difference between the two groups. Similarly, no significant differences were found in LAC or PaO_2_/FiO_2_ at 48 hours after surgery. Overall, this study did not detect significant differences between Group L and Group H. Furthermore, in cases where the FiO_2_ was 30% during the operation, 16% of the patients experienced intraoperative hypoxemia (SpO_2_ < 92%). This finding suggests that, for specific patient populations, an FiO_2_ of 30% during surgery may pose a safety risk, and it is necessary to take this situation into consideration in clinical practice.

As we know, perioperative hypoxia induces cellular oxidative stress by reducing the supply of tissue oxygen, consequently leading to organ damage, which is especially pronounced in elderly patients (Knight et al., 2022; Raberin et al., 2022). Oxygen serves as a routine perioperative support measure intended to prevent or treat hypoxemia and optimize tissue perfusion (Elfeky et al., 2022). Nevertheless, while hyperoxic exposure can elevate oxygen partial pressure, it may aggravate oxidative stress, mitochondrial dysfunction, neuronal damage and will increase the risk of atelectasis caused by absorption (Raberin et al., 2022; Hossain and Eckmann, 2023), and is linked to postoperative oxygenation deterioration (Choi et al., 2025). Although numerous studies have demonstrated that perioperative FiO_2_ of 30% is more beneficial than 80% in plain areas (Jiang et al., 2021; Douville et al., 2024; Elfeky et al., 2025), the arterial oxygen partial pressure at an altitude of 2500 meters above sea level is as much as 29% lower than that in plain areas (Richalet et al., 2024). In the special environment of the plateau, mechanically applying the conclusion that “30% is better than 80%” from the plain areas may not be applicable. Disregarding the partial pressures of carbon dioxide and water vapor in the atmosphere, based on Dalton’s law of partial pressures (West, 2016), at an altitude of 2500 meters, if 30% FiO_2_ is supplied, the inhaled oxygen partial pressure can only approximate the level of inhaled air (FiO_2_ 21%) in plain areas; whereas, when 50% FiO_2_ is provided, the inhaled oxygen partial pressure is slightly higher than that in plain areas with FiO_2_ 30%. Additionally, although the physiological structure and genes of residents in high-altitude areas have adapted to the hypoxic environment (Storz, 2021; Ferraretti et al., 2024), their basal arterial blood oxygen partial pressure and maximal oxygen uptake remain lower than those of residents in plain areas (Richalet et al., 2024). These factors may account for why perioperative FiO_2_ of 50% yields the optimal results for the elderly population in moderate altitude areas. Although there was insufficient evidence to substantiate the superiority or inferiority of intraoperative FiO_2_ of 30% and 80%, the findings of our research indicate that intraoperative FiO_2_ of 50% is superior to both 30% and 80%. This study may have determined a relatively ideal FiO_2_ of 50% for the specific population: elderly residents who have long-term residence in moderate-altitude areas and are scheduled to undergo laparoscopic radical surgery for gastrointestinal diseases.

Although no relevant literature regarding the impact of different FiO_2_ on Lung Ultrasound (LUS) was identified, this outcome aligns with our speculation and clinical observations. According to existing research, PPCs is associated with the accumulation of mechanical energy over time (Yoon et al., 2024). The duration of mechanical ventilation for the selected subjects was relatively prolonged (more than 3 hours), which may have contributed to the relatively high detection efficiency observed in this experiment. Furthermore, existing research has confirmed the correlation between LUS and PPCs (Boussier et al., 2024), which, from another perspective, further validates the statistical power of this study in detecting inter-group differences.

#### 4.4.2 The other outcomes

Based on the statistical results of CDYN, at the two time-points during the operation, Group M demonstrated the best performance, followed by Group L. Through a review of the existing literature, it was found that CDYN is associated with PEEP, body temperature, transpulmonary driving pressure, and the elastic recoil force of the lungs, and it represents a comprehensive manifestation of the pathological and physiological states of the lungs, including inflammatory status, alveolar structure, airway dynamics, and metabolic function.airway dynamics, and metabolic function (Stephan et al., 2021; Zhu et al., 2022; Ito et al., 2025). Upon a comprehensive review of the literature, no direct evidence was found to establish a link between CDYN and FiO_2_. Nevertheless, it is reasonable to postulate that an optimal FiO_2_ may enhance the overall pathological and physiological conditions of the lungs at the microscopic level. Another comprehensive indicator that reflects postoperative rehabilitation is the duration of postoperative hospital stay: Statistically, Group M demonstrated the best performance, whereas there was no significant disparity between Group L and Group H.

According to the statistical results, among the other outcomes, Group M still demonstrated the best performance, and this is consistent with our expectations.

## 5 Limitations

First, owing to the limitations of the design method, all the patients who were elimilated due to intraoperative SpO_2_ being less than 92% were from the Group L. This may have primarily affected the per-protocol sensitivity analysis results between the Group L and the Group H. Further studies and design enhancements are required in the future to address this deficiency. Second, routine oxygen inhalation preoperatively (3 L/min) and postoperatively (2 L/min for 48 hours) and permissive hypercapnia (PaCO_2_ 35–60 mmHg) during the operation would induce a significant number of physiological alterations, which could potentially impact pulmonary vascular tone, oxygenation index, and the progression of LUS. Future research designs should incorporate these factors into consideration. Third, this study was assessor-blinded. This may result in deviations in the experimental results. Consequently, in future research, double-blind or even triple-blind methods should be considered and implemented to the greatest extent possible. Fourth, the conclusion of this study indicates that 50% FiO_2_ is optimal, yet this is only in relation to 30% and 80%. Whether there exists a more suitable FiO_2_ requires further research for confirmation and to guide clinical strategies. Fifth, this study was solely focused on elderly patients in plateau areas with a pneumoperitoneum time of 3–4 hours. Whether it is applicable to other groups or other types of surgeries still needs further research for clarification. Sixth, during the implementation of the lung-protective ventilation strategy, all patients were given an empirical PEEP of 5 cm H_2_O during mechanical ventilation (Simon et al., 2021). However, titrating the optimal PEEP involves the measurement of multiple indicators such as lung compliance, electrical impedance tomography, esophageal pressure, and respiratory mechanics (Sarge et al., 2021; Sousa et al., 2025), which brought inconvenience to the study and may have had some impact on the observed indicators. Future research designs need to be further refined to attain the optimal PEEP without affecting the study results. Finally, the sample size was calculated based on the primary outcomes. Therefore, it is highly likely that our relatively small sample size underpowered the secondary outcomes. Large-scale randomized controlled trials should be conducted to address these limitations.

## 6 Conclusion

This study demonstrates that for elderly long-term residents at moderate altitudes, intraoperative FiO_2_ during laparoscopic surgery significantly influences postoperative outcomes, including LUS and recovery metrics such as PPCs and hospital stay. Among the three FiO_2_ levels tested (30%, 50%, and 80%), a setting of 50% was associated with the most favorable LUS and overall recovery, outperforming both 30% and 80%. No significant difference was observed between the 30% and 80% groups. Therefore, an intraoperative FiO_2_ of 50% appears to offer an optimal balance between adequate oxygenation and lung protection, potentially facilitating better postoperative recovery in this specific patient population.

## Data Availability

The raw data supporting the conclusions of this article will be made available by the authors, without undue reservation.
